# Serum lipidome screening in patients with stage I non-small cell lung cancer

**DOI:** 10.1007/s10238-019-00566-7

**Published:** 2019-07-01

**Authors:** Agnieszka Klupczynska, Szymon Plewa, Mariusz Kasprzyk, Wojciech Dyszkiewicz, Zenon J. Kokot, Jan Matysiak

**Affiliations:** 1 Department of Inorganic and Analytical Chemistry, Poznan University of Medical Sciences, Grunwaldzka 6 Street, 60-780 Poznan, Poland; 2 Department of Thoracic Surgery, Poznan University of Medical Sciences, Szamarzewskiego 62 Street, 60-569 Poznan, Poland

**Keywords:** Lung cancer, Lipidomics, Metabolomics, Phospholipids, Early diagnosis, Mass spectrometry

## Abstract

The ability of early lung cancer diagnosis is an unmet need in clinical practice. Lung cancer metabolomic analyses conducted so far have demonstrated several abnormalities in cancer lipid profile providing a rationale for further study of blood lipidome of the patients. In the present research, we performed a targeted lipidome screening to select molecules that show promise for early lung cancer detection. The study was conducted on serum samples collected from newly diagnosed, stage I non-small cell lung cancer (NSCLC) patients and non-cancer controls. A high-throughput mass spectrometry-based platform with confirmed interlaboratory reproducibility was used. The analyzed profile consisted of acylcarnitines, sphingomyelins, phosphatidylcholines and lysophosphatidylcholines. Among the assayed lipid species, the significant differences between NSCLC and non-cancer subjects were observed in the group of phosphatidylcholines (PC) and lysophosphatidylcholines (lysoPC), especially in the levels of lysoPC a C26:0; lysoPC a C26:1; PC aa C42:4; and PC aa C34:4. The metabolites mentioned above were used to create a multivariate classification model, which reliability was proved by permutation tests as well as external validation. Our study indicated choline-containing phospholipids as potential lung cancer markers. Further investigations of phospholipidome are crucial to better describe the shifts in metabolite composition occurring in lung cancer patients.

**Electronic supplementary material:**

The online version of this article (10.1007/s10238-019-00566-7) contains supplementary material, which is available to authorized users.

## Introduction

Due to the high incidence and mortality of lung cancer, there is a high demand for identification of cancer biomarkers that can contribute clinically relevant information. In both sexes combined, lung cancer is the main cause of cancer death worldwide (18.4% of the total cancer deaths) [1]. It is believed that the study of blood circulating markers would offer a chance to detect lung cancer in early stages. The results of many studies underscore the potential of metabolite analysis to uncover mechanisms of lung cancer and markers that could be useful in patients’ identification and discrimination. Based on results acquired from global metabolomics, it can be stated that the observed distinct metabolic profile in lung cancer patients is related to various classes of lipids and molecules involved in lipid metabolism [2]. Chen et al. [3] reported that sphingolipid metabolism was the top-altered metabolic pathway in lung cancer and proposed glycerophospho-N-arachidonoyl ethanolamine and sphingosine as biomarkers for lung cancer diagnosis and prognosis. Most of the differential metabolites identified by Li et al. [4] in serum profiling of lung cancer were associated with the perturbation of lipid metabolism, including free fatty acids, lysophosphatidylcholines and choline. Orbitrap-based global metabolic profiling revealed some putative lung cancer markers belonging to acylcarnitines [5]. Dong et al. [6] performed analysis of lysophosphatidylcholines in plasma samples of lung cancer patients and healthy donors using quadrupole time-of-flight mass spectrometry (Q-TOF), and they found abnormalities in five lysophosphatidylcholines species, including isomers. Based on lipid metabolite profiles obtained using Fourier transform ion cyclotron resonance mass spectrometry, a model of 7 metabolites consisting of 2 fatty acid derivatives, 4 lysophosphatidylcholines and sphingomyelin was developed, which allowed sample classification between lung cancer patients and healthy controls [7]. Downregulation of a few lysophosphatidylcholines was also demonstrated in MALDI-TOF-based serum lipid profiling [8]. The above-mentioned targeted research showed reliable and accurate metabolite identification. However, they did not produce data on analyte concentrations. Till now, few studies on quantitative analysis of lipidome of lung cancer patients have been reported showing promising results that should be further explored and validated [9, 10].

Lung cancer metabolomic analyses demonstrated several abnormalities in lipid profile providing a rationale for further study of blood lipidome of the patients. We performed a targeted, quantitative lipidomic profiling covering such metabolite classes as acylcarnitines, sphingomyelins, phosphatidylcholines and lysophosphatidylcholines in patients with non-small cell lung cancer and a control group. The identification of early lung cancer markers is a fundamental goal in studies aimed at searching for new diagnostic methods. Therefore, we applied rigorous inclusion criteria and enrolled only patients with stage I lung cancer. The applied methodology has already been used in metabolomic studies of other tumors: breast cancer [11], colorectal cancer [12], prostate cancer [13], pancreatic cancer [14], ovarian cancer [15] and bladder cancer [16]; however, it has not been applied to lung cancer research so far.

## Methodology

### Patient selection

Twenty patients with histopathologically confirmed lung cancer and twenty non-cancerous subjects (a control group) were recruited at the Department of Thoracic Surgery, Poznan University of Medical Sciences, Poland. All participants signed an informed written consent for this case–control study, which was approved by the Bioethics Committee of Poznan University of Medical Sciences (Decision no. 200/13). All lung cancer patients were diagnosed with non-small cell lung cancer (NSCLC) (Table 1). Patients pathological stages were determined at the Department of Thoracic Surgery, Poznan University of Medical Sciences using the TNM system (tumor size, node involvement, metastasis presence). Only patients with stage I cancer were selected for the study. Blood samples were collected before initiation of any cancer treatment. The mean age of lung cancer patients was 62 years, and 45% were female. Control subjects were age- and BMI-matched and consisted of the individuals of the same ethnic origin (Caucasians). The controls donated samples at the same time as cancer patients and met criteria for the absence of malignant disease, respiratory failure, hepatitis, or other diseases that can affect serum lipidome profile. Detailed information concerning the case–control set is shown in Table 1.

**Table 1:** Demographic and clinical characteristics of the study participants

Variable	Lung cancer patients	Healthy controls
Number of subjects, *n*	20	20
Age at recruitment, *y*		
Mean ± SD	62 ± 5	63 ± 6
Range	53–70	53–74
BMI, kg/m^2^		
Mean ± SD	26.2 ± 4.8	26.1 ± 3.5
Range	17.6–33.9	21.1–34.6
Gender		
%Male	55%	40%
Smoking status		
%Current smokers	60%	30%
Histologic subtype, *n*		
NSCLC, adenocarcinoma	9	
NSCLC, squamous cell carcinoma	11	
Clinical stage according to TNM classification, 7th ed, *n*		
IA	16	
IB	4	

### Sample collection and preparation

Blood samples were collected in the morning following an overnight fast using tubes with a clotting activator (S-Monovette system, Sarstedt, Nümbrecht, Germany). Then, serum was prepared according to a standardized protocol (centrifugation after 30 min at 4000 rpm for 5 min). The sera were aliquoted and stored at − 80 °C.

For serum metabolite profiling, AbsoluteIDQ p180 Kit (Biocrates Life Sciences AG, Innsbruck, Austria) was used. All assays were carried out on a 96-well plate according to the manufacturer’s recommended protocol. The sample preparation procedure was previously described in detail [17]. The reliability of the methodology was confirmed according to FDA guidance [18]. The kit allows the simultaneous determination of 145 lipid metabolites: 40 acylcarnitines, 15 sphingomyelins and 90 glycerophospholipids (14 lysophosphatidylcholines (lysoPC) and 76 phosphatidylcholines (PC)). The list of analyzed metabolites is contained in Online Resource.

### Instrumentation

Samples were analyzed in a random order using a triple quadrupole tandem mass spectrometer 4000 QTRAP (Sciex, Framingham, MA, USA) coupled with high-performance liquid chromatograph 1260 Infinity (Agilent Technologies, Santa Clara, CA, USA). The system was operated by the Analyst 1.5.2. software. A method based on flow injection analysis (FIA) and multiple reaction monitoring mode was applied. Injection volume was set at 20 μL. The remaining method parameters were set according to the Biocrates instructions. In-house verification of the validated methodology was performed with quality control (QC) samples at 3 concentration levels, which were provided in the kit and injected throughout the sequence. All of the measured lipid metabolites passed quality control. The measured metabolite concentrations were in agreement in with the established reference ranges indicating good accuracy. An average intra-assay coefficient of variation (CV) calculated from five repetitions of the QC sample was 4.4%, which proved low analytical variability. To additionally test the reliability of the lipid quantitation, we analyzed one serum sample in triplicate and the following CVs (%) were obtained: for acylcarnitines 13.4%, for lysoPC 11.1%, for PC aa 9.3%, for PC ae 11.4%, for sphingomyelins 9.2%.

### Data analysis

The MetIDQ software (Biocrates Life Sciences AG, Innsbruck, Austria) was used to conduct automated calculation of metabolite concentrations. Concentration values of all metabolites were reported in µM. For statistical analyses, metabolites determined in at least 80% of the samples were chosen. As a result, the lipid metabolite profile was restricted to a total of 104 metabolites (7 acylcarnitines, 15 sphingolipids and 81 glycerophospholipids) (Online Resource). Statistical tests were conducted using MetaboAnalyst 4.0 platform [19]. Before multivariate analyses, data were log-transformed and Pareto-scaled. Principal component analysis (PCA) was conducted to identify sample outliers and to assess the potential influence of different covariates on the obtained metabolic profiles. Before univariate tests, 6 samples (3 NSCLC and 3 controls) were randomly blinded and formed a validation set to assess the reliability of further developed multi-marker classification model. The performed univariate analyses included Wilcoxon rank-sum test, volcano plot and univariate receiver operating characteristic (ROC) curve analysis. Considering multi-testing problem, false discovery rate (FDR) was calculated in addition to the raw *p* value. The significance threshold for FDR was set to 0.05. The most differentiating features were selected to create the ROC curve-based model. Multivariate ROC curve analysis was performed based on the random forest algorithm, which uses random subsampling cross-validation. Finally, the created model was used to predict group for new samples (without group labels).

## Results

### PCA

The non-supervised multivariate PCA was applied to examine clustering or separation trends and find potential outliers. The obtained score plots indicated sample homogeneity (Online Resource). Partially separation of samples in line with the presence of lung cancer was discovered. Other tested variables (age, sex, BMI, smoking status) had no impact on sample clustering (Online Resource). Therefore, we identified no serious confounders in the dataset and found that disease status was the main factor responsible for the observed differences in the studied lipid metabolite profiles.

### Univariate tests

**Fig. 1:**
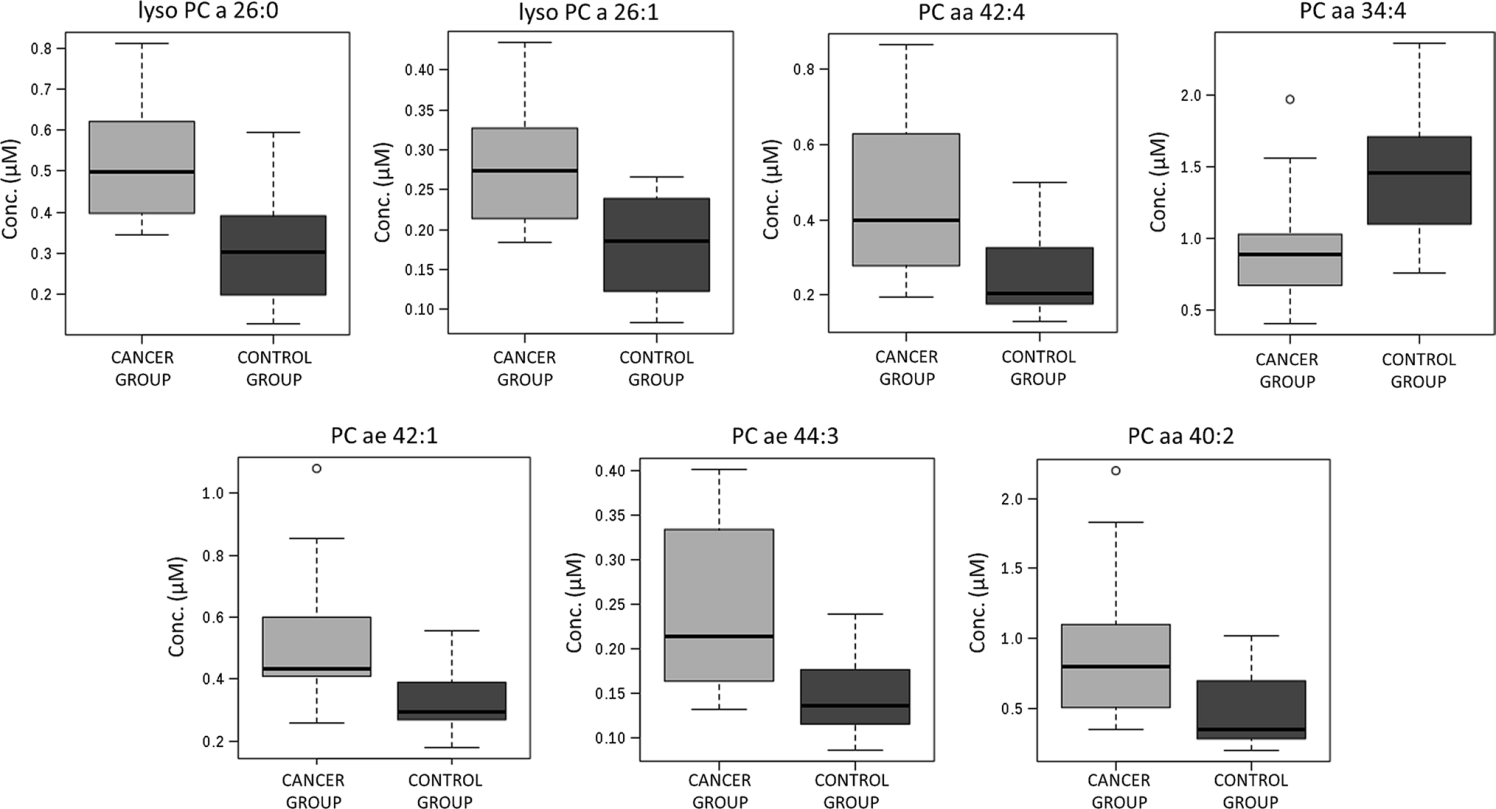
Box plots showing distributions of the selected lipid species (false discovery rate < 0.05 and fold change > 1.5) across the studied groups

In the comparison between patients with NSCLC and the control group, 11 out of 104 features from the obtained lipidome dataset had FDR values below 0.05. In the volcano plot, which is a combination of fold change and t tests, the following 7 variables met the set criteria (fold change threshold 1.5 and the FDR-corrected *p* value threshold 0.05): lysoPC a C26:0; lysoPC a C26:1; PC aa C42:4; PC aa C34:4; PC ae C42:1; PC ae C44:3; PC aa C40:2. Figure 1 contains boxplots of those metabolites showing that 6 compounds were significantly upregulated and 1 compound (PC aa C34:4) was significantly downregulated in samples from NSCLC patients compared with the non-cancer group. The results of univariate ROC curve analyses, a commonly used method for diagnostic biomarker evaluation, are demonstrated in Table 2. The highest AUC value, which is an indicator of the highest discriminating potential, was determined for lysoPC a C26:0.

**Table 2:** List of differentiating metabolites with their serum concentrations determined in the studied groups (mean ± SD, µM) and results from univariate statistics

Metabolite	Abbreviation	Lung cancer group^1^	Control group^1^	*p* value^2^	FDR^3^	Fold change^4^	AUC (95% CI)
Lysophosphatidylcholine acyl C26:0	lysoPC a C26:0	0.53 ± 0.14	0.31 ± 0.14	0.00011	0.01113	1.71	0.87 (0.73–0.96)
Lysophosphatidylcholine acyl C26:1	lysoPC a C26:1	0.28 ± 0.07	0.18 ± 0.06	0.00083	0.04337	1.52	0.84 (0.68–0.95)
Phosphatidylcholine diacyl C34:4	PC aa C34:4	0.93 ± 0.40	1.44 ± 0.43	0.00162	0.04529	0.65	0.82 (0.65–0.94)
Phosphatidylcholine diacyl C42:4	PC aa C42:4	0.46 ± 0.22	0.26 ± 0.12	0.00174	0.04529	1.79	0.81 (0.65–0.93)
Phosphatidylcholine acyl–alkyl C42:1	PC ae C42:1	0.69 ± 0.14	0.53 ± 0.13	0.00273	0.04729	1.57	0.80 (0.64–0.94)
Phosphatidylcholine acyl–alkyl C44:3	PC ae C44:3	0.52 ± 0.21	0.33 ± 0.11	0.00322	0.04789	1.58	0.80 (0.62–0.92)
Phosphatidylcholine diacyl C40:2	PC aa C40:2	0.24 ± 0.09	0.15 ± 0.04	0.00381	0.04904	1.88	0.80 (0.64–0.92)

^1^Values calculated from combined discovery and validation set

^2^Raw *p* value from Wilcoxon rank-sum test

^3^False discovery rate

^4^Calculated from the mean values of each group; comparison type: lung cancer group/control group

It should be noticed that the two study groups were balanced in terms of age and BMI, but not in terms of smoking status (Table 1). However, none of the significantly dysregulated metabolites discovered in our study is associated with smoking status according to the previous cohort studies [20, 21]. Therefore, we assume that the further proposed multi-metabolite model is not affected by nicotine-dependent potential biomarkers.

### Multivariate ROC curve analysis

**Fig. 2:**
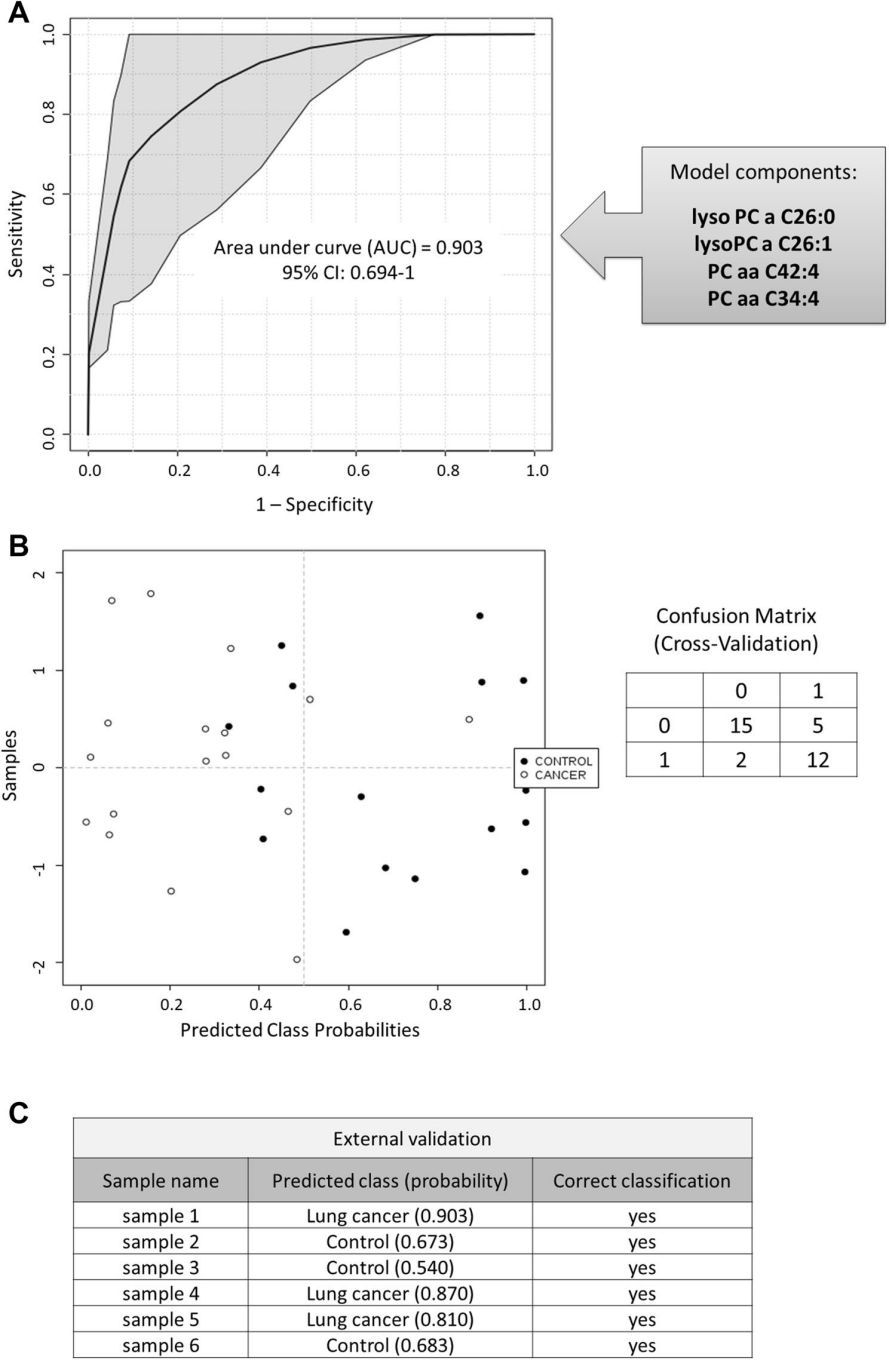
Performance of the created multivariate model composed of 4 lipid species: **a** the plot of the ROC curve for the model based upon its average cross-validation performance, **b** the plot of the predicted class probabilities for the samples using the proposed model, **c** blinded sample class prediction using the proposed model

Metabolites that exhibited the greatest differences between the studied groups according to the volcano plot (FDR-adjusted *p* value < 0.05 and fold change > 1.5) were selected to create the ROC curve-based multi-marker model. The model components were: lysoPC a C26:0; lysoPC a C26:1; PC aa C42:4; PC aa C34:4. The obtained AUC value of the model was based on cross-validated ROC curve. Therefore, it is more realistic for lung cancer prediction than in the case of univariate ROC curves (Fig. 2a, b). Nevertheless, the combination of 4 above-listed features yielded a greater AUC value than any single metabolite, providing the evidence that multi-metabolite classifier is more effective in sample distinguishing between the two studied groups. The performance of the multi-metabolite model was further evaluated by performing permutation tests (1000 repetitions). The calculated *p* value was 0.015, which indicates that the sample labels are not interchangeable and the model is significant. Additionally, we blinded a subset of 6 samples for extra validation of the model and all of the samples were correctly classified (Fig. 2c).

## Discussion

In the present study, we applied a wide targeted lipidome profiling, which yielded both a broad overview of the serum lipid composition of NSCLC patients as well as quantitation data. Among the assayed circulating metabolites, the major differences between cancer and non-cancer subjects were observed in the group of phosphatidylcholines and lysophosphatidylcholines (Table 2, Fig. 1). It should be emphasized that the observed abnormalities in the serum of NSCLC patients were found to be present at early disease stage (stage I).

Lipids play many roles at cellular and organismal levels, being the major structural components of biological membranes and energy storage entities. Moreover, lipids take part in signal transduction and can be broken down into bioactive lipid mediators, which regulate some carcinogenic processes, such as cell growth, proliferation and migration [22]. The analyzed lipidomic profile consisted of acylcarnitines, sphingomyelins, phosphatidylcholines and lysophosphatidylcholines. Levels of acylcarnitines, essential compounds for energy production, could mirror disturbances in fatty acid oxidation and organic acid metabolism in NSCLC patients [23, 24]. Although the previous untargeted metabolomic studies indicated that lung cancer marker candidates could be found in that metabolite class, no significant differences in the acylcarnitine profiles were observed in our study. However, the potential of short-chain acylcarnitines (carbon atoms < 10) should be still verified as they were present below LOQ in the majority of studied serum samples.

The significantly altered lipidome components found in the present study were choline-containing phospholipids. Five out of seven differentiating metabolites belong to phosphatidylcholines and the remaining belong to lysophosphatidylcholines (Table 2, Fig. 1). In a dominant pathway of phosphatidylcholine synthesis in humans, diacylglycerol combines with cytidine 5′-diphosphocholine (the Kennedy pathway). The first reaction of that process involves the phosphorylation of choline [15, 25]. It should be emphasized that both substrate (choline) and phosphatidylcholines were found to be associated with cancer development and progression. Overexpression and activation of choline cycle enzymes, such as choline kinase—a key enzyme in the biosynthesis of phosphatidylcholines, are emerging as a cancer biochemical hallmark [26–28]. Dysregulation of choline phospholipid metabolism was demonstrated in many cancer biomarker studies [29–31], including studies of lung cancer [32]. Analysis of malignant and matched non-malignant lung tissue revealed dramatic changes in phospholipid profiles of NSCLC [33]. Alterations in serum phospholipid profile of oncological patients shown in the current study were not so vast as those reported in lung tissue. In the comparison between NSCLC patients and the control group, 14% of choline-containing phospholipids quantified in the study were significantly (FDR-corrected *p* value < 0.05) discriminative (Online Resource).

Phosphatidylcholines, representing the most abundant glycerophospholipids in human plasma, are the main component of cell membranes as well as an important source of signaling molecules [34, 35]. The increased demand for membrane constituents leads to the upregulated synthesis of PCs in cancer cells [36]. In the present study, PC aa 42:4; PC ae 42:1; PC ae 44:3; and PC aa 40:2 were significantly increased in serum of NSCLC patients compared with non-cancer subjects. Elevated PCs with 40 or 42 carbon atoms in lung cancer tissue were previously reported by Marien et al. [33]. It indicates that the direction of change of PC levels in tumor tissue and blood of lung cancer patients is consistent. Our study revealed a trend for PCs to be increased in serum of lung cancer patients, which can relate to upregulation/activation of enzymes involved in PCs synthesis, i.e., choline kinase. Moreover, Lv et al. [37] demonstrated circulating levels of PC 40:1 and PC 40:4 as upregulated lipid molecular species that are specific for small cell lung cancer (SCLC). However, that conclusion was made based on very few samples from SCLC subjects. Addressing the question of whether the observed changes in lipidome of lung cancer patients are histologic-type specific requires large sample number and is an aim for future research.

The metabolites with the highest discriminating potential identified in our study belong to lysophosphatidylcholines (lysoPC 26:0 and lysoPC 26:1). The molecules of lysoPCs contain one fatty acyl group bonded to glycerol and are formed as a product of ester-bond hydrolysis of phosphatidylcholines catalyzed by phospholipase A2 [38]. This class of lipids represents approximately one tenth of the phospholipid fraction in humans [39] and function as an efficient cargo to provide fatty acids to tissues and organs (in a dynamic process of the Land’s cycle) [40, 41]. Apart from structural functions, lysoPCs have pro-inflammatory properties and are involved in signal transduction [42, 43]. Although our knowledge of lysophospholipid intracellular signaling is still growing, many questions remain to be answered. LysoPCs were identified among promising lung cancer marker candidates. Significantly increased blood levels of lysoPCs in lung cancer patients were shown by Li et al. [4], Guo et al. [7] and Dong et al. [44], whereas the downregulation of a few lysoPCs was demonstrated by Ros-Mazurczyk [8]. In the current study, elevated concentrations of lysoPCs were found among top-altered lipid profiles present in the blood of NSCLC patients. The highest discriminating ability in sample classification between NSCLC patients and controls had lysoPC 26:0 and lysoPC 26:1—lysoPCs with a very long acyl chain (*C* > 20). The increase in acyl chain length in cancer tissue was discovered as one of the most common traits of lung squamous cell carcinomas, found based on phospholipidome profiling [45]. The observed acyl chain elongation was accompanied by changes in the expression of acyl chain elongases (ELOVLs). Thus, it is suggested that inhibition of acyl chain elongation might be useful as a target for antineoplastic therapy in patients with squamous cell lung carcinomas. The high classification ability of lipid species with longer fatty acyl chains was also marked in the PC class in our study. Four out of 5 the most discriminative PCs belong to phospholipids with a large total number of acyl chain carbon atoms (≥ 36 carbon atoms in the two acyl chains together), which corresponds to the research of Marien et al. [45].

Lung cancer diagnosis needs refinement, and therefore, efforts should be taken to identify and develop new screening methods. The study presented here demonstrates that lysoPC a C26:0 had the highest discriminating ability in sample classification between patients with NSCLC and controls (Table 2). However, a question arises as to whether one molecule will have sufficient potential in NSCLC patients detection. Based on the results of previous cancer marker studies, it can be assumed that the most efficient sample classification will be obtained using multi-metabolite model. Therefore, we built a multivariate model consisting of 4 lipid species and tested it with a batch of blinded samples. The AUC of multivariate ROC curve was higher than that obtained for single compounds (Table 2, Fig. 2a) and the multi-compound classifier turned out to be robust enough to classify a new validation set of samples correctly (Fig. 2c). Therefore, our findings support the idea that the application of a combination strategy allows for better patients discrimination and shows promise for early lung cancer detection.

The present research has its own merits and limitations. The applied method covers a wide range of lipidome components providing data on concentrations of four classes of lipid metabolites (Online Resource). We used a high-throughput targeted metabolomic platform with proved interlaboratory reproducibility [46], which has been previously used to catalog other disease states and identify potential biomarker profiles. Another strength of the research is patient selection and restriction to early NSCLC cases (stage I). Therefore, our findings are not biased by metabolic profiles of subjects with an advanced tumor and present the potential of lipid metabolites in early NSCLC detection. The application of the strict inclusion criteria decreased the number of patients, and the next step should involve the inclusion of a multicenter group of subjects to better estimate the accuracy of the developed model in early lung cancer detection. The reliability of the proposed classifier was proved by permutation tests as well as a small test set of samples for external validation. Another limitation of our research is related to identification difficulties and technical limitation. Lipidomics is one of the most demanding fields of metabolomics due to the huge variety of lipid species. In our study, the measured metabolites are described by a number of carbon atoms in both fatty acyl chains together and number of double bonds without their exact position, where PC aa and PC ae abbreviations denote diacyl phosphatidylcholine and acyl-alkyl phosphatidylcholine, respectively. The amazing complexity of the human serum lipidome is a challenge, but the continuous technological development, mainly in mass spectrometry, enables the quantification of lipids with increasing depth and accuracy [47].

## Concluding remarks

In the present research, we conducted a lipidome screening to select molecules that show promise for early lung cancer detection. As quantitative metabolomic data are particularly desirable in studies on searching for new cancer markers, we applied a targeted method covering a wide range of lipidome components. Our study indicated choline-containing phospholipids as a promising source of lung cancer markers, especially lysoPC aC26:0; lysoPC a C26:1; PC aa C42:4; PC aa C34:4. It is anticipated that the use of lipidomics will continue to increase in lung cancer biomarker studies and enhances the ability of researchers to study dysregulation of phospholipid metabolism in cancer patients.

## Electronic supplementary material

Below is the link to the electronic supplementary material.

Supplementary material 1 (DOCX 361 kb)
