# Supplementary material for: Serum lipidome screening in patients with stage I non-small cell lung cancer
Source: Clin Exp Med. 2019 Jul 1;19(4):505–13. doi: 10.1007/s10238-019-00566-7 (PMC6797644; doi:10.1007/s10238-019-00566-7)
Supplement: Supplementary file 1 — Supplementary material 1 (DOCX 361 kb) [file 10238_2019_566_MOESM1_ESM.docx]

Electronic Supplementary Material

*Clinical and Experimental Medicine*

**Serum lipidome screening in patients with stage I non-small cell lung cancer**

Agnieszka Klupczynska^1^, Szymon Plewa^1^, Mariusz Kasprzyk^2^, Wojciech Dyszkiewicz^2^, Zenon J. Kokot^1^, Jan Matysiak^1^

^1^ Department of Inorganic and Analytical Chemistry, Poznan University of Medical Sciences, Grunwaldzka 6 Street, 60-780 Poznan, Poland

^2^ Department of Thoracic Surgery, Poznan University of Medical Sciences, Szamarzewskiego 62 Street, 60-569 Poznan, Poland

Corresponding author:

Agnieszka Klupczynska, Ph.D.

Department of Inorganic and Analytical Chemistry,

Poznan University of Medical Sciences,

6 Grunwaldzka Street, 60-780 Poznan, Poland

phone: 0048 61 854 66 10, fax: 0048 61 854 66 09

email address: aklupczynska@ump.edu.pl

ORCID 0000-0002-5028-1408

ESM 1. List of analyzed metabolites.

| Class | Analyte abbreviations* | Number  of metabolites analyzed | Number  of analytes quantified in serum samples |
| --- | --- | --- | --- |
| Carnitine and acylcarnitines | C0; C2; C3; C3-OH; C3:1; C3-DC (or C4-OH); C4; C4:1; C5; C5-M-DC; C5-OH (or C3-DC-M); C5:1; C5:1-DC; C5-DC (or C6-OH); C6 (or C4:1-DC); C6:1; C7-DC; C8; C9; C10; C10:1; C10:2; C12; C12-DC; C12:1; C14; C14:1; C14:1-OH; C14:2; C14:2-OH; C16; C16-OH; C16:1; C16:1-OH; C16:2; C16:2-OH; C18; C18:1; C18:1-OH; C18:2 | 40 | 8 |
| Lysophosphatidylcholines (lysoPC) | lysoPC a C14:0; lysoPC a C16:0; lysoPC a C16:1; lysoPC a C17:0; lysoPC a C18:0; lysoPC a C18:1; lysoPC a C18:2; lysoPC a C20:3; lysoPC a C20:4; lysoPC a C24:0; lysoPC a C26:0; lysoPC a C26:1; lysoPC a C28:0; lysoPC a C28:1 | 14 | 12 |
| Phophatidylcholines  (PC) | PC aa C24:0; PC aa C26:0; PC aa C28:1; PC aa C30:0; PC aa C30:2; PC aa C32:0; PC aa C32:1; PC aa C32:2; PC aa C32:3; PC aa C34:1; PC aa C34:2; PC aa C34:3; PC aa C34:4; PC aa C36:0; PC aa C36:1; PC aa C36:2; PC aa C36:3; PC aa C36:4; PC aa C36:5; PC aa C36:6; PC aa C38:0; PC aa C38:1; PC aa C38:3; PC aa C38:4; PC aa C38:5; PC aa C38:6; PC aa C40:1; PC aa C40:2; PC aa C40:3; PC aa C40:4; PC aa C40:5; PC aa C40:6; PC aa C42:0; PC aa C42:1; PC aa C42:2; PC aa C42:4; PC aa C42:5; PC aa C42:6; PC ae C30:0; PC ae C30:1; PC ae C30:2; PC ae C32:1; PC ae C32:2; PC ae C34:0; PC ae C34:1; PC ae C34:2; PC ae C34:3; PC ae C36:0; PC ae C36:1; PC ae C36:2; PC ae C36:3; PC ae C36:4; PC ae C36:5; PC ae C38:0; PC ae C38:1; PC ae C38:2; PC ae C38:3; PC ae C38:4; PC ae C38:5; PC ae C38:6; PC ae C40:1; PC ae C40:2; PC ae C40:3; PC ae C40:4; PC ae C40:5; PC ae C40:6; PC ae C42:0; PC ae C42:1; PC ae C42:2; PC ae C42:3; PC ae C42:4; PC ae C42:5; PC ae C44:3; PC ae C44:4; PC ae C44:5; PC ae C44:6 | 76 | 69 |
| Sphingomyelins  (SM) | SM (OH) C14:1; SM (OH) C16:1; SM (OH) C22:1; SM (OH) C22:2; SM (OH) C24:1; SM C16:0; SM C16:1; SM C18:0; SM C18:1; SM C20:2; SM C22:3; SM C24:0; SM C24:1; SM C26:0; SM C26:1 | 15 | 15 |
| Total |  | 163 | 104 |

^*^ Lipid side chain composition is presented as Cx:y, where “x” corresponds to the number of carbons and “y” corresponds to the number of double bonds. Letter “a” denotes the presence of ester bond in glycerol moiety, letter “e” denotes the presence of ether bond in glycerol moiety. Letters “aa” indicate diacyl-phosphatidylcholines, letters “ae” indicate acyl-alkyl-phosphatidylcholines.

ESM 2. Metabolite concentrations determined in the studied serum samples (medians along with lower- upper quartiles; µM).

| Metabolite | Lung cancer patients  (n=20) | Control group  (n=20) |
| --- | --- | --- |
| C0 | 43.9 (37.6;52.1) | 41.0 (33.3;45.9) |
| C10:1 | 0.135 (0.104;0.183) | 0.121 (0.073;0.153) |
| C12:1 | 0.164 (0.150;0.247) | 0.164 (0.145;0.201) |
| C14:1 | 0.118 (0.099;0.150) | 0.104 (0.083;0.170) |
| C14:2 | 0.041 (0.035;0.054) | 0.036 (0.027;0.05) |
| C18:1 | 0.136 (0.115;0.193) | 0.162 (0.133;0.199) |
| C18:2 | 0.041 (0.034;0.054) | 0.052 (0.041;0.063) |
| C2 | 7.02 (4.97;7.90) | 6.61 (5.14;9.05) |
| lysoPC a C16:0 | 79.8 (68.2;97.6) | 94.0 (80.9;107.5) |
| lysoPC a C16:1 | 2.61 (1.93;3.25) | 3.14 (2.59;3.54) |
| lysoPC a C17:0 | 5.75 (4.91;7.13) | 6.96 (5.92;8.07) |
| lysoPC a C18:0 | 24.2 (19.8;28.4) | 28.5 (23.6;34.8) |
| lysoPC a C18:1 | 15.9 (12.0;20.0) | 20.3 (14.9;23.8) |
| lysoPC a C18:2 | 15.5 (11.5;18.2) | 21.3 (14.8;26.9) |
| lysoPC a C20:3 | 1.66 (1.55;2.17) | 2.04 (1.80;2.64) |
| lysoPC a C20:4 | 5.93 (4.87;7.27) | 7.57 (6.64;8.50) |
| lysoPC a C26:0 | 0.506 (0.422;0.618) | 0.321 (0.206;0.438) |
| lysoPC a C26:1 | 0.265 (0.207;0.322) | 0.183 (0.135;0.243) |
| lysoPC a C28:0 | 0.472 (0.415;0.601) | 0.333 (0.233;0.489) |
| lysoPC a C28:1 | 0.651 (0.561;0.750) | 0.534 (0.474;0.660) |
| PC aa C28:1 | 2.03 (1.52;2.38) | 2.38 (1.86;2.68) |
| PC aa C30:0 | 7.68 (6.36;8.27) | 8.38 (7.36;8.90) |
| PC aa C30:2 | 0.876 (0.751;1.005) | 0.964 (0.811;1.29) |
| PC aa C32:0 | 12.1 (10.9;13.1) | 11.6 (10.8;12.6) |
| PC aa C32:1 | 12.0 (10.0;15.7) | 12.5 (9.88;16.6) |
| PC aa C32:2 | 1.38 (0.868;1.66) | 2.01 (1.83;2.82) |
| PC aa C32:3 | 0.641 (0.487;0.706) | 0.623 (0.569;0.764) |
| PC aa C34:1 | 181 (164;210) | 174 (156;198) |
| PC aa C34:2 | 247 (223;266) | 246 (233;273) |
| PC aa C34:3 | 9.75 (8.71;12.3) | 11.4 (9.51;12.9) |
| PC aa C34:4 | 0.903 (0.676;1.03) | 1.45 (1.15;1.71) |
| PC aa C36:0 | 4.74 (4.08;5.41) | 5.13 (4.66;5.61) |
| PC aa C36:1 | 42.2 (37.9;49.5) | 46.5 (42.2;53.7) |
| PC aa C36:2 | 146 (124;163) | 155 (140;173) |
| PC aa C36:3 | 97.0 (87.6;104) | 99.0 (88.7;112) |
| PC aa C36:4 | 135 (120;149) | 141 (119;171) |
| PC aa C36:5 | 16.5 (12.2;21.7) | 20.2 (17.3;28.5) |
| PC aa C36:6 | 0.750 (0.582;1.01) | 0.999 (0.785;1.19) |
| PC aa C38:0 | 3.06 (2.59;3.38) | 3.54 (2.64;4.09) |
| PC aa C38:1 | 0.904 (0.653;1.21) | 0.765 (0.610;0.973) |
| PC aa C38:3 | 43.1 (37.3;51.8) | 44.8 (37.4;50.6) |
| PC aa C38:4 | 80.0 (71.7;105) | 93.1 (72.8;111) |
| PC aa C38:5 | 40.3 (33.1;46.2) | 47.6 (39.0;53.0) |
| PC aa C38:6 | 59.1 (50.9;75.9) | 71.4 (61.1;82.5) |
| PC aa C40:2 | 0.787 (0.494;1.05) | 0.361 (0.281;0.665) |
| PC aa C40:3 | 1.00 (0.786;1.28) | 0.694 (0.585;1.02) |
| PC aa C40:4 | 3.38 (2.95;4.28) | 3.34 (2.82;3.79) |
| PC aa C40:5 | 7.90 (6.24;10.1) | 9.13 (7.21;9.54) |
| PC aa C40:6 | 22.8 (17.3;28.1) | 23.9 (21.4;31.0) |
| PC aa C42:0 | 0.521 (0.435;0.686) | 0.550 (0.393;0.720) |
| PC aa C42:1 | 0.291 (0.230;0.371) | 0.272 (0.221;0.343) |
| PC aa C42:2 | 0.321 (0.266;0.414) | 0.287 (0.216;0.334) |
| PC aa C42:4 | 0.389 (0.265;0.585) | 0.207 (0.176;0.356) |
| PC aa C42:5 | 0.368 (0.341;0.500) | 0.337 (0.313;0.411) |
| PC ae C30:0 | 0.359 (0.316;0.432) | 0.425 (0.299;0.497) |
| PC ae C30:1 | 0.495 (0.407;0.573) | 0.494 (0.467;0.563) |
| PC ae C32:1 | 2.12 (1.69;2.48) | 2.27 (1.74;2.47) |
| PC ae C32:2 | 0.909 (0.781;1.04) | 1.01 (0.888;1.08) |
| PC ae C34:0 | 1.27 (1.17;1.49) | 1.66 (1.31;1.95) |
| PC ae C34:1 | 7.13 (6.06;8.05) | 7.90 (6.28;9.20) |
| PC ae C34:2 | 6.77 (4.96;7.78) | 7.66 (6.39;9.36) |
| PC ae C34:3 | 5.57 (3.99;7.16) | 5.90 (5.69;7.24) |
| PC ae C36:0 | 1.46 (1.32;1.75) | 1.24 (0.988;1.50) |
| PC ae C36:1 | 8.58 (7.24;10.5) | 8.63 (6.52;9.44) |
| PC ae C36:2 | 7.81 (6.58;8.79) | 9.53 (8.75;10.8) |
| PC ae C36:3 | 5.60 (4.61;6.75) | 6.65 (5.51;7.88) |
| PC ae C36:4 | 10.7 (9.13;13.0) | 12.8 (10.9;16.4) |
| PC ae C36:5 | 20.1 (17.8;22.2) | 21.3 (18.5;23.6) |
| PC ae C38:0 | 2.04 (1.76;2.48) | 2.21 (1.84;2.53) |
| PC ae C38:1 | 2.45 (1.97;3.05) | 1.75 (1.14;2.75) |
| PC ae C38:2 | 2.99 (2.58;4.21) | 2.22 (1.79;3.37) |
| PC ae C38:3 | 7.32 (5.55;9.42) | 4.85 (3.75;7.72) |
| PC ae C38:4 | 10.2 (8.45;11.4) | 11.6 (9.83;13.1) |
| PC ae C38:5 | 11.6 (9.88;13.8) | 13.8 (11.0;15.9) |
| PC ae C38:6 | 5.57 (4.78;6.66) | 7.34 (5.64;8.13) |
| PC ae C40:1 | 1.12 (0.96;1.36) | 1.19 (1.10;1.32) |
| PC ae C40:2 | 2.34 (2.02;2.80) | 2.31 (1.87;2.58) |
| PC ae C40:3 | 3.39 (2.16;4.63) | 1.27 (1.08;3.30) |
| PC ae C40:4 | 3.11 (2.42;4.37) | 2.33 (1.97;3.44) |
| PC ae C40:5 | 4.34 (3.34;5.61) | 3.72 (3.20;4.44) |
| PC ae C40:6 | 3.40 (3.00;4.12) | 4.22 (3.42;5.28) |
| PC ae C42:1 | 0.429 (0.385;0.562) | 0.297 (0.268;0.417) |
| PC ae C42:2 | 0.586 (0.538;0.801) | 0.571 (0.524;0.617) |
| PC ae C42:3 | 0.828 (0.723;1.18) | 0.793 (0.749;0.864) |
| PC ae C42:4 | 0.559 (0.444;0.894) | 0.481 (0.337;0.716) |
| PC ae C44:3 | 0.209 (0.163;0.301) | 0.142 (0.122;0.184) |
| PC ae C44:4 | 0.434 (0.384;0.562) | 0.418 (0.380;0.501) |
| PC ae C44:5 | 1.45 (1.23;1.84) | 1.53 (1.26;2.04) |
| PC ae C44:6 | 0.930 (0.804;1.23) | 1.13 (0.812;1.27) |
| SM (OH) C14:1 | 2.89 (1.61;3.60) | 3.38 (2.64;4.64) |
| SM (OH) C16:1 | 2.41 (1.79;2.98) | 3.01 (2.35;3.35) |
| SM (OH) C22:1 | 10.2 (9.15;11.0) | 12.3 (10.3;13.6) |
| SM (OH) C22:2 | 9.66 (8.09;11.2) | 11.4 (9.77;12.1) |
| SM (OH) C24:1 | 1.22 (1.09;1.31) | 1.22 (1.11;1.40) |
| SM C16:0 | 90.0 (80.7;105) | 89.3 (81.6;98.4) |
| SM C16:1 | 13.1 (11.4;14.7) | 12.6 (11.4;14.5) |
| SM C18:0 | 22.5 (17.1;23.8) | 21.7 (18.0;25.5) |
| SM C18:1 | 11.7 (8.68;14.1) | 10.0 (8.88;13.7) |
| SM C20:2 | 0.839 (0.751;1.052) | 0.978 (0.810;1.05) |
| SM C22:3 | 6.81 (5.60;7.73) | 8.29 (6.88;10.9) |
| SM C24:0 | 22.0 (20.3;24.5) | 23.1 (19.6;25.8) |
| SM C24:1 | 58.3 (51.1;69.5) | 54.4 (48.9;63.4) |
| SM C26:0 | 0.182 (0.162;0.198) | 0.167 (0.134;0.213) |
| SM C26:1 | 0.504 (0.343;0.543) | 0.462 (0.375;0.552) |


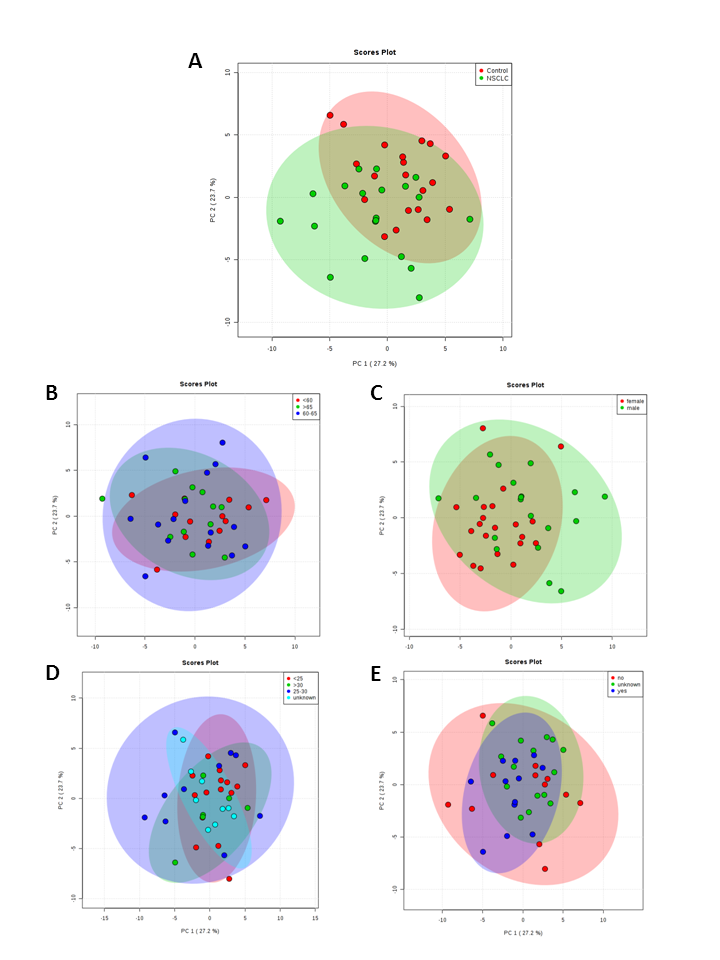


ESM 3. Principal component analysis (PCA) scores plots. Samples are divided into groups according to: (A) presence of non-small cell lung cancer: NSCLC and control group; (B) age: <60, 60-65, >65; (C) sex: male and female; (D) body mass index (BMI): <25, 25-30, >30, unknown; (E) smoking: yes, no, unknown.


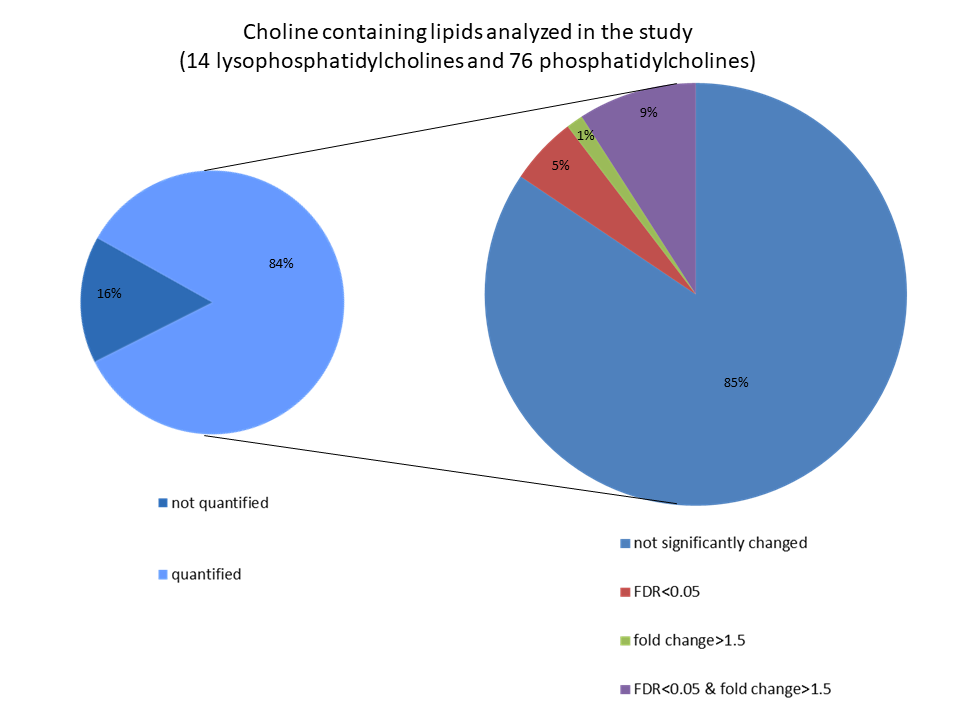


ESM 4. The percentage of the choline-containing lpipids that were significantly changed in NSCLC patients. The applied methodology allows for determination of 90 choline-containing lipids, among which 11 were significantly discriminative (FDR-corrected *p*-value<0.05).
